# A COMPARISON OF SUBJECTIVE COGNITIVE DECLINE AND RELATED LIMITATIONS AMONG TRANSGENDER COMMUNITIES IN THE US

**DOI:** 10.1093/geroni/igac059.808

**Published:** 2022-12-20

**Authors:** Ethan Cicero, Michael Goodman, Lisa Barnes, Molly Perkins, Jason Flatt, Vin Tangpricha

**Affiliations:** Emory University, Atlanta, Georgia, United States; Emory University, Atlanta, Georgia, United States; Rush Alzheimer’s Disease Center, Rush University Medical Center, Chicago, Illinois, United States; Emory University School of Medicine, Atlanta, Georgia, United States; University of Nevada, Las Vegas, School of Public Health, Las Vegas, Nevada, United States; Emory University, Atlanta, Georgia, United States

## Abstract

**Background:**

The transgender population is composed of subgroups that are diverse in gender identity (e.g., transgender women[TW], transgender men[TM], nonbinary[NB] individuals). Compared to cisgender adults, transgender adults are more likely to report subjective cognitive decline (SCD). It remains unclear if SCD prevalence and related limitations vary by transgender subgroups.

**Methods:**

2015-2020 Behavioral Risk Factor Surveillance System data, representing 38 U.S. states that assessed SCD (confusion/memory loss happening more often/getting work over previous 12months) and gender identity were used to examine differences in SCD prevalence and SCD-related limitations by transgender subgroups, TW(n=442), TM(n=298), and NB(n=183). Age-adjusted odds ratios (OR) along with 95% confidence intervals (CI) were calculated to investigate group differences in SCD prevalence. Separate analyses compared SCD-related limitations, demographics, and health across groups among participants reporting SCD.

**Results:**

SCD prevalence was highest among NB(21.3%), followed by TW(16.3%) and TM(14.1%). After accounting for age, subgroup differences remained; odds of SCD were 1.6x higher among TW compared to TM (CI:1.1–2.4, p=0.012). Among those with SCD, TW were less likely to receive help they needed with day-to-day activities when compared to TM (OR=7.9; CI:0.1–0.2, p< 0.001) and NB (OR=5.0; CI:0.1–0.4, p=0.001); and TW were more likely to be deaf (OR=4.2; CI:1.7–10.1, p=0.002) and have asthma (OR=2.8; CI:1.4–5.7, p=0.005) when compared to NB adults. No other differences were found.

**Conclusion:**

Health and social inequities are not uniformly experienced across transgender subgroups, and it is important to understand how these factors impact the brain health of TW, TM, and NB adults.

